# Evaluation of organic fractions of municipal solid waste as renewable feedstock for succinic acid production

**DOI:** 10.1186/s13068-020-01708-w

**Published:** 2020-04-15

**Authors:** Eleni Stylianou, Chrysanthi Pateraki, Dimitrios Ladakis, María Cruz-Fernández, Marcos Latorre-Sánchez, Caterina Coll, Apostolis Koutinas

**Affiliations:** 1 Department of Food Science and Human Nutrition, Agricultural University of Athens, Iera Odos 75, 118 55 Athens, Greece; 2 IMECAL SA, Carretera Carlet, 74, L’Alcudia, 46250 Valencia, Spain

**Keywords:** Succinic acid, Organic fraction of municipal solid waste, Continuous fermentation, *Actinobacillus succinogenes*, Bioprocess, Techno-economic evaluation

## Abstract

**Background:**

Despite its high market potential, bio-based succinic acid production experienced recently a declining trend because the initial investments did not meet the expectations for rapid market growth. Thus, reducing the succinic acid production cost is imperative to ensure industrial implementation.

**Results:**

Succinic acid production has been evaluated using hydrolysates from the organic fraction of municipal solid waste (OFMSW) collected from MSW treatment plants. A tailor-made enzymatic cocktail was used for OFMSW hydrolysate production containing up to 107.3 g/L carbon sources and up to 638.7 mg/L free amino nitrogen. The bacterial strains *Actinobacillus succinogenes* and *Basfia succiniciproducens* were evaluated for succinic acid production with the latter strain being less efficient due to high lactic acid production. Batch *A. succinogenes* cultures supplemented with 5 g/L yeast extract and 5 g/L MgCO_3_ reached 29.4 g/L succinic acid with productivity of 0.89 g/L/h and yield of 0.56 g/g. Continuous cultures at dilution rate of 0.06 h^−1^ reached 21.2 g/L succinic acid with yield of 0.47 g/g and productivity of 1.27 g/L/h. Downstream separation and purification of succinic acid was achieved by centrifugation, treatment with activated carbon, acidification with cation exchange resins, evaporation and drying, reaching more than 99% purity. Preliminary techno-economic evaluation has been employed to evaluate the profitability potential of bio-based succinic acid production.

**Conclusions:**

The use of OFMSW hydrolysate in continuous cultures could lead to a minimum selling price of 2.5 $/kg at annual production capacity of 40,000 t succinic acid and OFMSW hydrolysate production cost of 25 $/t sugars.

## Background

In the period 2013–2017, the average annual MSW production in EU was 482 kg/capita and 245.3 million t, with the top five producing countries being Germany (20.9%), France (14%), UK (12.7%), Italy (12.1%) and Spain (8.7%) [1]. The composition of MSW varies among EU countries consisting mainly of organic material (25–48%), paper (16–51%), plastics (0.8–23.7%), glass/ceramic (4.5–16%), metals (2–8.8%) and textiles and other components (3.1–32.2%) [1]. The main management practices of MSW in EU countries are material recycling, incineration, composting and landfilling. Energy recovery from MSW in EU-28 was on average 24.7% for the period 2013–2017 [1], with the top 5 countries being Norway (53.7%), Denmark (53.2%), Sweden (51.8%), Finland (50%) and Estonia (48.7%). In the EU, the organic fraction of municipal solid waste (OFMSW) or municipal biowaste is defined as the biodegradable garden and park waste, food and kitchen waste from households, offices, restaurants, wholesale, canteens, caterers and retail premises and comparable waste from food processing plants according to the EU Directive 2018/851 [2]. The biowaste fraction constitutes 30–40% (or even 20–80%) of the total MSW [3]. The carbohydrate content of OFMSW is 30–60% depending on the origin of the waste and the region among other factors. The OFMSW has been evaluated for the production of polyhydroxyalkanoates (PHAs), biogas and biohydrogen via mixed cultures [4–6] as well as the production of bio-based fuels and platform chemicals, such as bioethanol, lactic acid and succinic acid, via single strain fermentations [7–9].

Besides the traditional food and pharmaceutical markets, succinic acid is used for the production of bio-based plasticizers, poly(butylene succinate), polyester polyols, coatings, lubricants, resins and personal care products. Succinic acid can be used as platform chemical for the production of 1,4-butanediol, tetrahydrofuran and γ-butyrolactone among other chemicals [10]. Despite the high potential of succinic acid as platform chemical and the high prospects for industrial growth, in recent years, the growth of bio-based succinic acid production is declining because the four initial industrial producers (i.e., Myriant, BioAmber, Reverdia and Succinity) have faced severe competition with low petroleum prices due to increased supply of shale oil and gas that reduced the price of petrochemicals [11–13]. Although BioAmber closed the production plant in Sarnia (Canada), the company LCY Biosciences that purchased the facility will restart succinic acid production [14]. The facilities of Myriant and Succinity are currently idle, with Reverdia’s succinic acid production facility being the only operational one [13]. The market price of bio-based succinic acid (2.94 $/kg) is higher than the market price of the combined bio- and fossil-based succinic acid (2.5 $/kg) [10]. The cost of bio-based succinic acid production could be reduced through the utilization of crude renewable resources that can support its production at high capacities to benefit from economies of scale. Within a circular economy context, the OFMSW constitutes a cheap and widely available feedstock the carbohydrate content of which could be relatively easily hydrolysed for succinic acid production.

Succinic acid production has been studied using various types of food waste collected from biowaste bins provided by municipalities containing also other organic materials [9], catering services [15] or specific types of food waste, such as waste bread [16]. Babaei et al., [9] used biowaste hydrolysates collected at source in bins as fermentation feedstock in *Basfia succiniciproducens* cultures for the production of 36 g/L succinic acid concentration with yield lower than 0.4 g/g and productivity lower than 0.3 g/L/h. Li et al. [15] reported the production of 18.9 g/L succinic acid concentration with yield of 0.38 g/g and productivity of 0.25 g/L/h when a genetically engineered *Yarrowia lipolytica* strain was cultivated in mixed food waste hydrolysate. The hydrolysis of food waste or biowaste fractions has been mainly carried out using either enzyme consortia produced on-site (e.g., via solid state fungal fermentation) [17] or commercial enzyme mixtures [9, 18]. The succinic acid production cost could be reduced further by combining the utilization of low-cost feedstock with continuous fermentations that leads to high productivities [19, 20].

The production of succinic acid has never been evaluated using the OFMSW separated in central MSW management facilities, which is currently the predominant worldwide management practice. The combination of industrially optimized tailor-made enzyme mixtures for OFMSW hydrolysis with succinic acid production via fermentation has also not been evaluated in literature-cited publications. Furthermore, continuous cultures for the production of succinic acid from OFMSW hydrolysates have not been evaluated in comparison to fed-batch cultures. This study has evaluated all the aforementioned aspects including the estimation of the threshold of OFMSW pretreatment cost in order to achieve a cost-competitive process for the production of succinic acid crystals of high purity. The widely studied wild-type bacterial strains *Actinobacillus succinogenes* and *B. succiniciproducens* have been used in this study to provide a comparison basis with literature-cited succinic acid production efficiencies achieved by these strains on various crude hydrolysates.

## Results and discussion

### OFMSW composition

Table 1 presents the composition of different OFMSW samples used to prepare the OFMSW hydrolysates. Non-biodegradable materials contained in OFMSW (glass, stones, plastics, sand, etc.) can cause serious technological problems in industrial facilities (clogging, erosion in equipment) and reduce the performance of biological processes. Unsorted biowaste obtained via mechanical sorting processing using mixed MSW from household bins contained higher contents of inert material (non-biodegradable) and ash as well as lower moisture content than sorted biowaste. The glucan content, representing both cellulose and starch, is slightly higher in sorted biowaste (ca. 40%, db). Most of glucan originates from cellulose since the obtained starch content is 4–5.3% in all cases. The xylan content was lower than 5% (db) in sorted biowaste, while it was higher than 5% (db) in unsorted biowaste. The pectin content, originating mainly from fruit waste, is lower (10.1–12.19%, db) in unsorted biowaste than sorted biowaste (15.87–18.25%, db). The protein was higher (8.75–10.15%, db) in sorted biowaste, while the fat content (4.59–5.86%, db) was higher in unsorted biowaste. The lignin content varies (5.64–11.02%, db) among all OFMSW samples. This low content of lignin in municipal biowaste is advantageous compared with other lignocellulosic wastes (typically > 25% in woods).

**Table 1:** Characterization of OFMSW samples from real MSW treatment plant

Components (%, dry basis)	Sorted biowaste		Unsorted biowaste	
	Spring/summer	Autumn/winter	Spring/summer	Autumn/winter
Total solids	31.36 ± 0.5	16.14 ± 0.5	43.3 ± 4	51.61 ± 0.9
Ash	6.25 ± 0.3	7.59 ± 0.7	19.2 ± 0.7	23.07 ± 1.1
Fats and waxes	1.57 ± 0.9	3.15 ± 1.2	4.59 ± 0.7	5.86 ± 0.8
Water solubles	36.20 ± 0.4	42.56 ± 0.3	27.9 ± 2.2	15.59 ± 1.9
Pectin	15.87 ± 2.9	18.25 ± 0.4	10.1 ± 2.0	12.19 ± 0.8
Lignin	9.47 ± 0.6	8.00 ± 1.2	5.64 ± 0.8	11.02 ± 1.2
Glucan (cellulose and starch)	39.64 ± 5.4	39.83 ± 0.5	36.57 ± 2.9	25.06 ± 0.3
Xylan	0.2 ± 0.01	1.92 ± 0.5	5.14 ± 0.2	8.67 ± 0.4
Protein	10.15 ± 0.2	8.75 ± 0.5	7.4 ± 0.7	7.0 ± 0.4
Starch	4.71 ± 1.5	5.31 ± 0.9	4.36 ± 0.3	4.17 ± 0.7
Inert materials (%, wet basis)	4%	5%	25%	36%

The primary components of sorted municipal solid waste are cellulose (45%), hemicellulose (9%) and lignin (10%) [21]. Garcia et al. [22] characterized different fractions of biodegradable municipal solid waste (meat, fish, fruit and vegetable, restaurant and household waste). Dry matter, ash content and crude protein in the different fractions varied between 11.9–59.0%, 4.9–21.8% and 11.6–57.0% (on a dry basis, db), respectively [22].

### OFMSW hydrolysate production

OFMSW mechanical pretreatment and enzymatic hydrolysis was performed by IMECAL S.A. with tailor-made enzymatic cocktails provided by Novozymes. The enzyme content and the corresponding enzyme activities are confidential and cannot be mentioned in this publication. Glucan content (including both cellulose and starch) varied in the range of 25–40% (db) and xylan content ranged from 0.2 to 8.7% (db). Glucan (cellulose and starch) hydrolysis conversion yield was 75% (w/w) and xylan conversion yield was around 12.5% (w/w). Table 2 presents the variation in the composition of different batches of OFMSW hydrolysates produced in this study. The total dry weight measured after hydrolysis was in the range of 114.17–118.81 g/L in all cases. In the liquid fraction, the total sugar concentration in OFMSW hydrolysate ranged between 31.2 and 107.3 g/L with 70.7–81.3% glucose (25.4–75.9 g/L), 7.1–12.6% xylose (3.95–7.6 g/L) and 0.3 to 14.4% fructose (0.1–15.5 g/L). Glycerol, sucrose, galactose, arabinose, mannose concentrations were less than 5% of the total sugar content. Free amino nitrogen (FAN) and inorganic phosphorus (IP) concentrations in the liquid fraction of OFMSW ranged between 203.6–638.7 mg/L and 100.6–553 mg/L, respectively.

**Table 2:** Composition of liquid and solid fractions of OFMSW hydrolysates

Components of solid and liquid OFMSW hydrolysate fractions	Range
Total dry weight (g/L)	114.17–118.81

Liquid fraction of OFMSW hydrolysate	
pH	4.36–5.18
Free amino nitrogen (mg/L)	203.6–638.7
Inorganic phosphorus (mg/L)	100.6–553
Kjeldahl protein content (%)	1.72–2.86
Sugars (g/L)	31.2–107.3
Sucrose	0.25–3.58
Glucose	25.4–75.9
Xylose	3.95–7.6
Galactose	0.2–1.4
Arabinose	0.5–1.5
Mannose	0.2–0.8
Fructose	0.1–15.5
Glycerol	0.63–1
Total organic acids (g/L)	12.2–22.5
Lactic acid	10.7–18.6
Acetic acid	1.5–3.7

Solid fraction of OFMSW hydrolysate (% dry basis)	
Ash content	5.7–25
Protein content	7.0–19.85
Lipid content	6.8–7.6
Extractives	33.23–33.34
Lignin	16.92–27.39

Cellulose	
Glucan	9.07–9.46

Hemicellulose	
Xylan	6.64–6.91
Galactan	0.91–0.94
Mannan	4.46–4.52
Arabinan	–

Significant lactic acid concentrations (10.7–18.6 g/L) and lower acetic acid concentrations (1.5–3.7 g/L) were detected in all OFMSW hydrolysates. These organic acids were present since the beginning of the hydrolysis indicating contamination of OFMSW despite the origin of biowaste streams. No organic acid production or bacterial growth was observed during hydrolysis due to the aseptic conditions used. Furfural and 5-hydroxymethylfurfural were not detected in OFMSW hydrolysates. This was expected as these inhibitory compounds can be generated from the degradation of xylose and glucose under intensive chemical treatment.

In the solid fraction that remained after OFMSW hydrolysis, the ash (5.7–25%, db) and protein (7–19.85%, db) contents varied at a wide range depending on the origin of OFMSW. The lipid content was 6.8–7.6% (db). Water and ethanol soluble extractives were ca. 33% (db). Lignin, cellulose and hemicellulose ranged from 16.92–27.39%, 9.07–9.46% and 12.01–12.37% (db), respectively. Hemicellulose consisted of xylan, galactan and mannan fractions.

### Batch fermentations

Table 3 presents succinic acid production via fermentation using *B. succiniciproducens* and *A. succinogenes* at different initial total carbon source concentrations using either commercial carbon sources or OFMSW hydrolysate. Fermentations using the OFMSW hydrolysate enhanced the productivity of both microorganisms compared to the commercial medium, with 52% increase on average in the case of *B. succiniciproducens* and 32% in the case of *A. succinogenes* (Table 3). The highest succinic acid concentration that was observed in the case of *B. succiniciproducens* fermentations was 37.1 g/L in OFMSW hydrolysate and in the case of *A. succinogenes* was 37.9 g/L both in OFMSW hydrolysate and commercial substrate (Table 3).

**Table 3:** Fermentation efficiency of *B. succiniciproducens* and *A. succinogenes* batch cultures carried out at three initial concentrations of commercial carbon sources and OFMSW hydrolysate

Initial and consumed carbon sources (g/L)^a^	Medium	Succinic acid (g/L)	Yield (g/g)^a^	Productivity (g/L/h)	By-product: succinic acid (g/g)
*Basfia succiniciproducens*					
30 (27.8)	Commercial	12.5	0.45	0.44	1.19
30 (31.3)	OFMSW	14.4	0.46	0.64	0.90
50 (51.7)	Commercial	26.9	0.52	0.45	0.66
50 (50.6)	OFMSW	26.8	0.53	0.85	0.88
80 (81.1)	Commercial	35.7	0.44	0.46	0.56
80 (78.9)	OFMSW	37.1	0.47	0.56	0.68
*Actinobacillus succinogenes*					
30 (30.8)	Commercial	15.4	0.5	0.6	0.71
30 (30.2)	OFMSW	15.7	0.52	0.52	0.71
50 (47.1)	Commercial	27.3	0.58	0.49	0.20
50 (52.7)	OFMSW	29.4	0.56	0.89	0.31
80 (78.2)	Commercial	37.9	0.54	0.45	0.21
80 (77.3)	OFMSW	37.9	0.5	0.57	0.31

^a^The initial total carbon source concentration is ± 5% of the indicated value, while the consumed carbon source is presented in the brackets; succinic acid to sugars conversion yield has been calculated based on the quantity (g) of succinic acid produced during fermentation and the quantity (g) of total sugars added in the bioreactor

The by-product to succinic acid ratio decreased with increasing initial total carbon source concentration for both *A. succinogenes* and *B. succiniciproducens* in both fermentation media. *B. succiniciproducens* resulted in a decrease of by-product to succinic acid ratio at around 50% in the case of glucose-based fermentations and up to 25% in the case of OFMSW hydrolysate. *A. succinogenes* by-product to succinic acid ratio decreased up to 84% in the case of glucose and up to 65% in the case of OFMSW hydrolysate (Table 3). The major difference between the two microorganisms lies on the fact that lactic acid production from *B. succiniciproducens* occurs throughout fermentation. When OFMSW hydrolysates were used, lactic acid and acetic acid were present at the beginning of fermentation and they have been excluded from the ratios presented in Table 3.

**Fig. 1:**
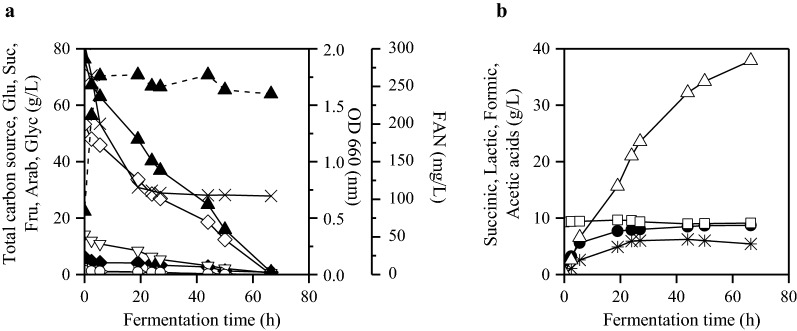
Carbon source consumption, optical density (OD) and metabolic product synthesis during batch fermentation of *A. succinogenes* using OFMSW hydrolysate at initial total carbon source concentration of 80 g/L. **a** Total carbon source (filled triangle), glucose (open rhombus), sucrose (filled rhombus), fructose (open inverted triangle), arabinose (right pointing triangle), glycerol (open circle), OD (660 nm)  . **b** FAN (times), succinic acid (open triangle), lactic acid (open square), formic acid (star) and acetic acid (filled circle)

Figure 1 presents experimental results of batch fermentation carried out with *A. succinogenes* using OFMSW hydrolysate at initial total sugar concentration of 80 g/L. The final succinic acid concentration was 37.9 g/L with yield of 0.5 g/g and productivity of 0.57 g/L/h. The initial FAN concentration using both the synthetic medium and the OFMSW hydrolysate was in the range of 251–285 mg/L in all batch fermentations. FAN consumption occurred in the first 24 h and remained constant (at around 100 mg/L) until the end of fermentation (Fig. 1a).

Babaei et al. [9] carried out batch fermentations with *B. succiniciproducens* cultivated in a hydrolysate from the organic fraction of household kitchen waste (the sugars contained 85% glucose and 15% xylose) with CO_2_ supply from either MgCO_3_ or raw biogas. Succinic acid production efficiency of around 5.5 g/L with yield 0.39 g/g and 3.8 g/L with yield 0.25 g/g, respectively [9]. *A. succinogenes* has been employed for succinic acid production using deacetylated dilute acid pretreated corn stover hydrolysate leading to the production of 42.8 g/L succinic acid with yield of 0.74 g/g and maximum productivity of 1.27 g/L/h [23]. Glucose-rich food waste have also been used for the production of succinic acid in batch cultures by an engineered *Yarrowia lipolytica* strain resulting in 31.7 g/L succinic acid concentration with yield of 0.52 g/g and productivity of 0.60 g/L/h [24]. Waste bread and bakery waste hydrolysates have also been used as raw materials for the production of succinic acid. Fermentation with bakery waste by *A. succinogenes* resulted in 47.3 g/L succinic acid with a yield of 1.16 g/g glucose and productivity of 1.12 g/L h [16]. Cake and pastry hydrolysates resulted in 24.8 g/L (yield 0.8 g/g, productivity 0.79 g/L/h) and 31.7 g/L (yield 0.67 g/g, productivity 0.87 g/L/h) succinic acid concentration, respectively [25].

From a techno-economic viewpoint, it is crucial to identify the initial carbon source concentration in bioreactor cultures leading to the highest succinic acid concentration, yield and productivity. Results from this study clearly demonstrate that OFMSW hydrolysates at a 50 g/L initial carbon source concentration resulted in the highest productivity and yield for both microorganisms. Liu et al. [26] reported that growth inhibition of *A. succinogenes* was observed at 50 g/L initial glucose concentration. Salvachua et al. [23] carried out batch fermentations at different initial glucose concentrations (40–100 g/L) with the highest yield (0.72 g/g) achieved at initial glucose concentration of 60 g/L. *A. succinogenes* can tolerate up to 143 g/L glucose and cell growth was completely inhibited when glucose concentration was higher than 158 g/L [27]. However, significant decrease of yield and prolonged lag phase were observed when glucose concentration was higher than 100 g/L [27]. Using a xylose-based medium, the initial inhibitory sugar concentration was around 50 g/L for both *A. succinogenes* and *B. succiniciproducens* [28].

Subsequent fed-batch fermentations on OFMSW hydrolysates were carried out with *A. succinogenes* at ca. 50 g/L initial carbon source concentration, where yield, productivity and by-product to succinic acid ratio were optimum. *B. succiniciproducens* was not selected due to significant lactic acid production during fermentation.

### Fed-batch bioreactor fermentations

Table 4 presents the succinic acid production efficiency of *A. succinogenes* in fed-batch fermentations using OFMSW hydrolysate. Evaluation of the effect of different initial MgCO_3_ concentration (5, 10, 20 g/L) resulted in moderate improvement of succinic acid production efficiency at increasing concentrations. The highest succinic acid concentration (34.8 g/L), yield (0.6 g/g) and productivity (0.79 g/L/h) were achieved when 20 g/L MgCO_3_ concentration was used. The production of metabolic by-products was slightly reduced when MgCO_3_ concentration was increased to 10 g/L. Magnesium ions act as a cofactor for the key enzyme phosphoenolpyruvate (PEP) carboxykinase [29] and carbonate ions (HCO_3_^−^, CO_3_^2−^) are a pool of additional CO_2_ [30]. CO_2_ in the form of gas or carbonate salts have been previously investigated by McKinlay et al. [31] resulting in increased succinic acid concentration in favor of by-product accumulation due to the suppression of *OAAdec* and *Maldec* towards pyruvate. According to Brink et al. [32], *A. succinogenes* is able to metabolize formate to CO_2_ and H_2_O towards the production of NADH. As a result, the generation of NADH contributes to enhanced succinic acid production. However, significant enhancement of succinic acid production efficiency was not observed in this study at high MgCO_3_ concentrations. For this reason, the lowest MgCO_3_ concentration (5 g/L) was used in subsequent fermentations in order to minimize raw material cost and environmental impact.

**Table 4:** Fermentation efficiency of *A. succinogenes* fed-batch cultures carried out using OFMSW hydrolysate

Initial and consumed carbon sources (g/L)^a^	Nitrogen source (g/L)	MgCO_3_ (g/L)	Succinic acid (g/L)	Yield (g/g)^a^	Productivity (g/L/h)	By-product: succinic acid (g/g)
50 (57.9)	5 g/L CSL	5	28.7	0.50	0.41	0.37
50 (68.4)	5 g/L YE	5	34.3	0.50	0.75	0.59
50 (55.1)	–	5	31.1	0.56	0.62	0.30
50 (57.1)	–	10	31.7	0.56	0.72	0.25
50 (58.4)	–	20	34.8	0.60	0.79	0.25

^a^Consumed carbon source is presented in the brackets; succinic acid to sugars conversion yield has been calculated based on quantity (g) of succinic acid produced during fermentation and the quantity (g) of total sugars added in the bioreactor

**Fig. 2:**
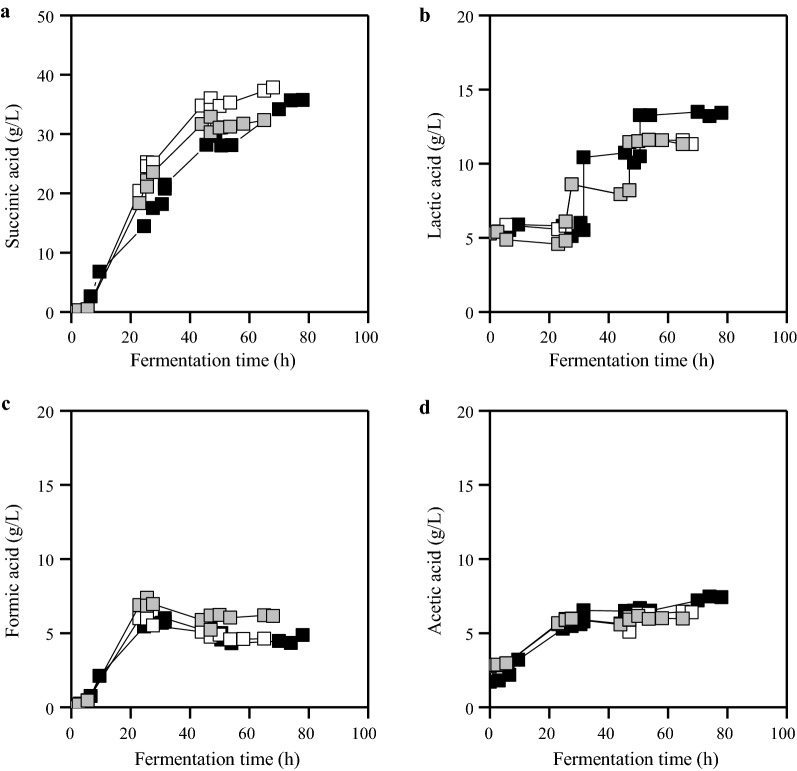
Succinic acid (**a**), formic acid (**c**) and acetic acid (**d**) production and lactic acid (**b**) accumulation in fed-batch fermentations of *A. succinogenes* using OFMSW hydrolysate supplemented with three different initial MgCO_3_ concentrations, specifically 5 g/L (filled black square), 10 g/L (filled grey square) and 20 g/L MgCO_3_ (open square)

Figure 2 presents the metabolic product formation and lactic acid accumulation during fed-batch cultures carried out with different initial MgCO_3_ concentrations. Lactic acid (Fig. 2) accumulation due to feeding of OFMSW hydrolysate reached concentrations close to 10 g/L when higher MgCO_3_ concentrations were used. The lactic acid concentration in batch cultures with 50 g/L initial carbon source concentration from OFMSW hydrolysates was constant (10 g/L) during fermentation resulting in higher productivity (0.89 g/L/h) and similar yield (0.56 g/g) (Table 3) as compared to fed-batch cultures carried out with 5 g/L and 10 g/L initial MgCO_3_ concentration.

**Fig. 3:**
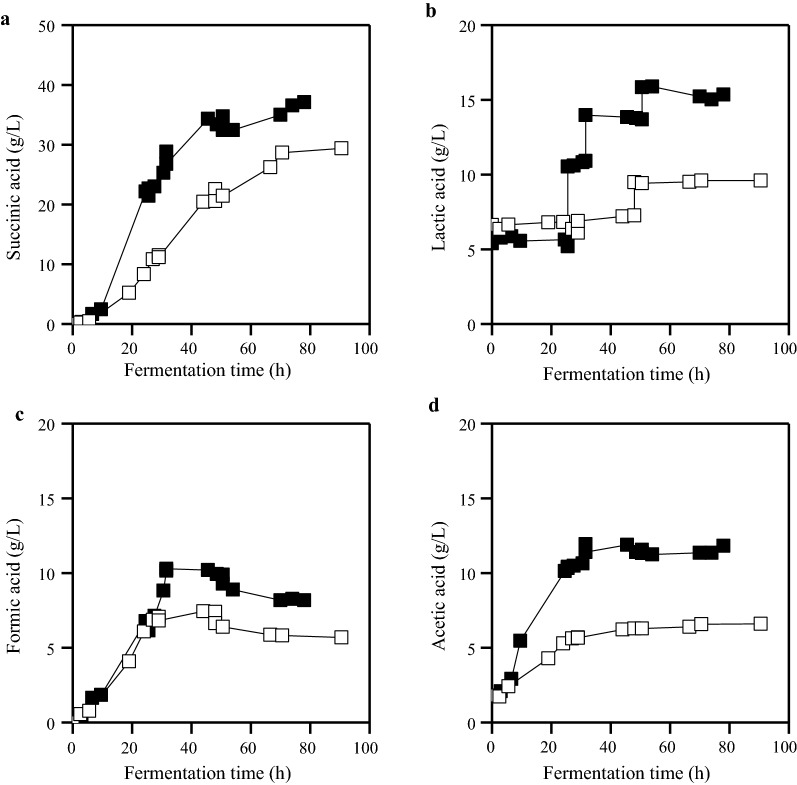
Succinic acid (**a**), formic acid (**c**) and acetic acid (**d**) production and lactic acid (**b**) accumulation in fed-batch fermentations of *A. succinogenes* using OFMSW hydrolysate supplemented with 5 g/L MgCO_3_ and either 5 g/L yeast extract (filled square) or 5 g/L CSL (open square)

Yeast extract (YE) and corn steep liquor (CSL) supplementation was also evaluated in fed-batch fermentations using OFMSW hydrolysate supplemented with 5 g/L MgCO_3_ (Fig. 3). Addition of 5 g/L CSL resulted in 28.7 g/L of succinic acid with a yield of 0.5 g/g and a productivity of 0.41 g/L/h. Yeast extract supplementation (5 g/L) resulted in significantly higher succinic acid concentration (34.3 g/L) and productivity (0.75 g/L/h). However, the utilization of yeast extract resulted in the highest by-product to succinic acid ratio (0.59) among all fed-batch fermentations presented in Table 4.

Yeast extract enhances cells growth and succinic acid production. Liu et al. [26] reported that yeast extract results in slightly higher succinic acid concentration than CSL in *A. succinogenes* CGMCC1593 cultures. CSL derived from corn refining has been widely employed as the sole nitrogen source resulting in high succinic acid concentrations (47.4 g/L) [33]. Chen et al. [34] reported the production of 35.5 g/L succinic acid in *A. succinogenes* cultures carried out with 50 g/L glucose concentration and spent yeast cell hydrolysate, with a glucose utilization of 95.2%.

### Continuous fermentation

**Fig. 4:**
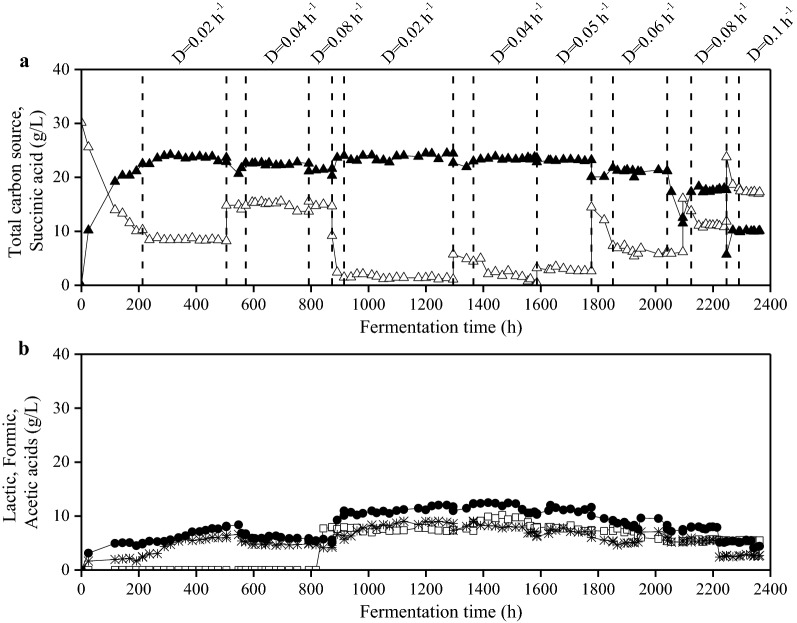
Succinic acid (filled triangle), acetic acid (filled circle) and formic acid (star) production as well as total sugars (open triangle) and lactic acid (open square) accumulation during continuous fermentation of *A. succinogenes* at different dilution rates using either commercial carbon source (until 900 h) or OFMSW hydrolysate (until 2400 h)

Figure 4 presents carbon source consumption and metabolic product accumulation during continuous fermentation of *A. succinogenes*. The continuous culture was carried out with glucose as carbon source until 900 h, since the major sugar fraction in OFMSW hydrolysate is glucose (73.2%). OFMSW hydrolysate was used as feeding solution from 900 h until 2400 h. At 237 h, biofilm formation was developed and thus steady-state conditions were established. Three dilution rates (0.02, 0.04 and 0.08 h^−1^) were used with glucose and 6 dilution rates (0.02, 0.04, 0.05, 0.06, 0.08 and 0.1 h^−1^) were used with OFMSW hydrolysate. Figure 4 shows that steady state was achieved within 2–4 days depending on the dilution rate used.

**Fig. 5:**
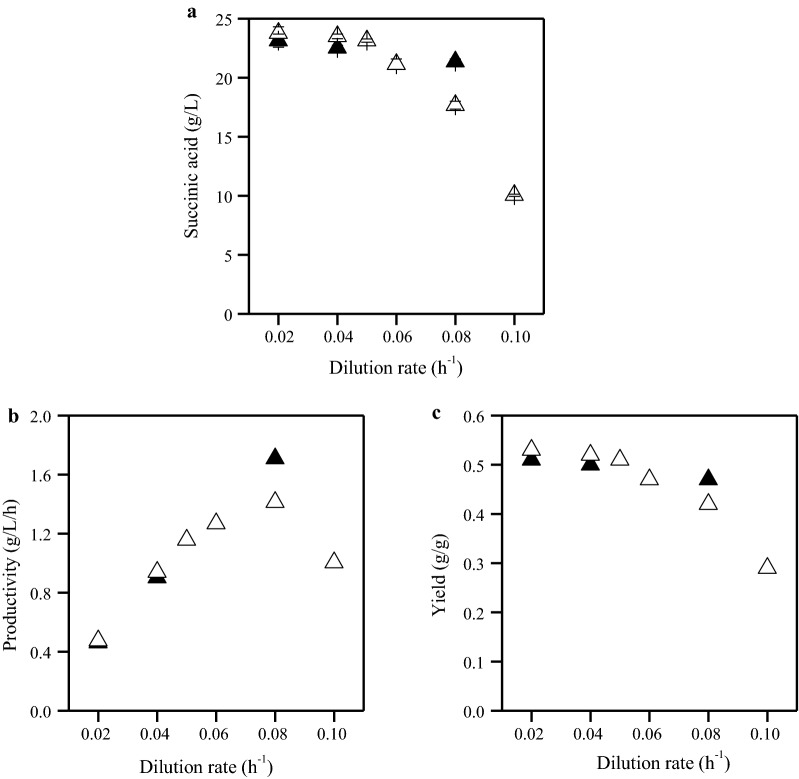
Succinic acid concentration (**a**), productivity (**b**) and yield (**c**) achieved during *A. succinogenes* continuous cultures using OFMSW hydrolysate (unfilled symbols) and synthetic medium (filled symbols) at different dilution rates. Data represent the average values of the steady states at each dilution rate. Succinic acid to sugars conversion yield has been calculated based on quantity (g) of succinic acid produced during fermentation and the quantity (g) of total sugars added in the bioreactor

Using glucose and a dilution rate of 0.02 h^−1^, the average succinic acid production was 23.1 g/L with a yield of 0.51 g/g (Fig. 5). Succinic acid concentration slightly decreased at increasing dilution rates. Specifically, it was 22.5 g/L (0.50 g/g yield) and 21.2 g/L (0.47 g/g yield) at dilution rates of 0.04 h^−1^ and 0.08 h^−1^, respectively. Increased dilution rates resulted in increased productivity, namely 0.46 g/L/h, 0.9 g/L/h and 1.71 g/L/h at dilution rates of 0.02 h^−1^, 0.04 h^−1^ and 0.08 h^−1^, respectively.

OFMSW hydrolysate resulted in an average succinic acid production of 23.8 g/L with a yield of 0.53 g/g at dilution rate 0.02 h^−1^ (Fig. 5). Succinic acid concentration (23.5–23.1 g/L) and yield (0.52–0.51 g/g) were stable at 0.04 h^−1^ and 0.05 h^−1^. Higher dilution rates resulted in decreasing succinic acid concentration and yield. Specifically, succinic acid concentration was 21.2 g/L, 17.7 g/L and 10 g/L at dilution rates of 0.06 h^−1^, 0.08 h^−1^ and 0.1 h^−1^, respectively. The productivity increased at increasing dilution rates, up to 0.08 h^−1^, with an average productivity of 0.48 g/L/h, 0.94 g/L/h, 1.16 g/L/h, 1.27 g/L/h, and 1.41 g/L/h at dilution rates of 0.02 h^−1^, 0.04 h^−1^, 0.05 h^−1^, 0.06 h^−1^ and 0.08 h^−1^, respectively. Significant decrease in succinic acid production efficiency was observed at 0.1 h^−1^ due to cell wash-out.

Formic and acetic acid production was observed throughout continuous fermentation carried out either with glucose or OFMSW hydrolysate (Fig. 4b). In the latter case, lactic acid accumulation was observed due to its presence in the OFMSW hydrolysate. Acetic acid concentrations produced by *A. succinogenes* were 7.1 g/L, 5.9 g/L and 5.4 g/L at dilution rates 0.02, 0.04 and 0.08 h^−1^, respectively, in glucose-based medium. The respective formic acid concentrations were 5.7 g/L, 4.8 g/L and 4.4 g/L. The by-products to succinic acid ratio in glucose at dilution rate of 0.02 h^−1^ was 0.55 g/g, while at increasing dilution rates (0.04 and 0.08 h^−1^) by-products to succinic acid ratio was around 0.46 g/g.

When *A. succinogenes* was cultivated on OFMSW hydrolysate, the acetic acid concentration was always higher than formic acid concentrations at all dilution rates. The highest acetic acid concentrations were observed at dilution rates of 0.02 h^−1^ (9.9 g/L) and 0.04 h^−1^ (9.3 g/L). The highest concentration of total by-products for *A. succinogenes* was observed at dilution rate of 0.02 h^−1^ (12.7 g/L in glucose and 18 g/L in OFMSW hydrolysate). By-products to succinic acid ratio using OFMSW hydrolysate as feeding was 0.76 g/g, 0.73 g/g, 0.71 g/g, 0.62 g/g, 0.63 g/g and 0.6 g/g at dilution rates of 0.02 h^−1^, 0.04 h^−1^, 0.05 h^−1^, 0.06 h^−1^, 0.08 h^−1^ and 0.1 h^−1^, respectively.

**Fig. 6:**
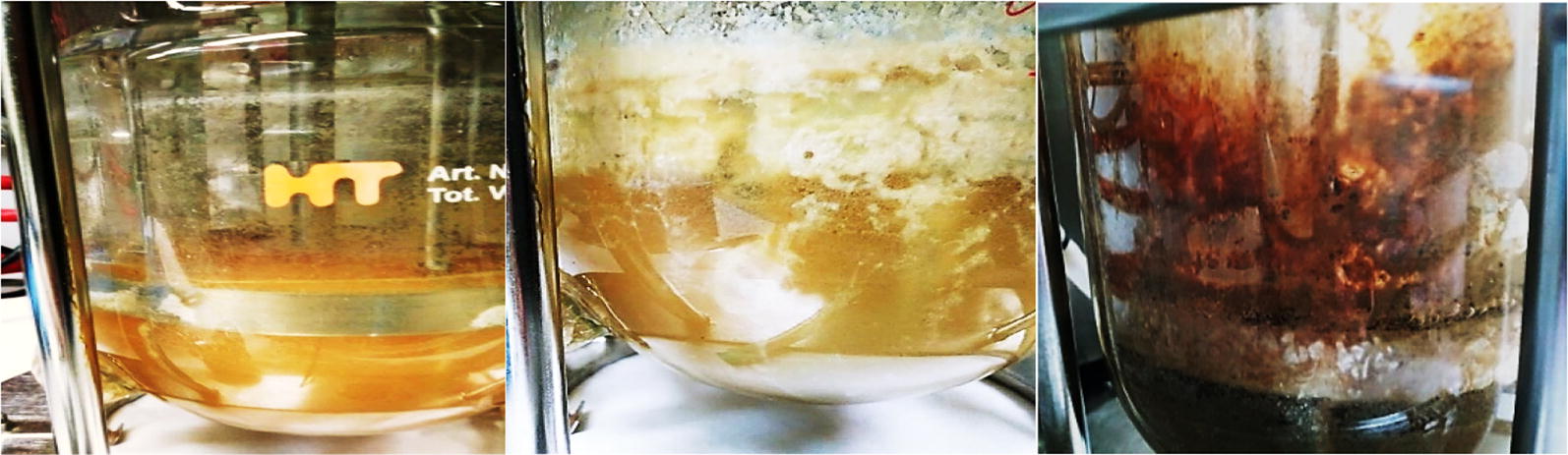
Wall growth and biofilm formation in continuous *A. succinogenes* culture at the beginning of fermentation (left), during feeding with commercial sugars (middle) and during feeding with OFMSW hydrolysate (right)

Biofilm formation occurred on the wall and the mechanical parts of the bioreactor during continuous fermentation (Fig. 6). Continuous *A. succinogenes* cultures under prolonged operation period result in biofilm formation [35–37]. Ladakis et al. [19] carried out continuous *A. succinogenes* fermentation using synthetic xylose as carbon source leading to succinic acid concentration of 24 g/L with yield 0.6 g/g at 0.02 h^−1^. Higher yield (0.77 g/g) and succinic acid concentration (26.4 g/L) was achieved in continuous cultures of immobilized *A. succinogenes* cells using synthetic xylose at dilution rate 0.1 h^−1^ [20]. Continuous *A. succinogenes* cultures carried out on glucose at dilution rate 0.11 h^−1^ in a biofilm reactor packed with Poraver^®^ beads led to 29.5 g/L succinic acid concentration with productivity of 3.24 g/L/h and yield of 0.9 g/g [38]. Ferone et al. [39] reported continuous succinic acid fermentation of *A succinogenes* in a packed-bed biofilm reactor leading to 43 g/L succinic acid concentration at dilution rate 0.5 h^−1^ with glucose conversion of 88%. Continuous *A. succinogenes* cultures on glucose conducted in a fibrous-bed bioreactor led to 55.3 g/L succinic acid concentration with 0.8 g/g yield and 2.77 g/L/h production at dilution rate 0.05 h^−1^ [40].

Various crude hydrolysates from various biomass sources have been used for the production of bio-based succinic acid. Continuous fermentation on spent sulfite liquor resulted in 19.2 g/L succinic acid concentration with yield 0.48 g/g at dilution rate 0.02 h^−1^, while the highest productivity was 0.68 g/L/h at dilution rate 0.04 h^−1^ [19]. Brandfield et al. achieved succinic acid concentration of 39.6 g/L via immobilized *A. succinogenes* continuous cultures carried out on corn stover hydrolysate [20].

### Process feasibility evaluation

A preliminary techno-economic evaluation was carried out considering the optimum succinic acid production efficiency achieved in batch and continuous *A. succinogenes* cultures using OFMSW hydrolysate as feedstock. The batch fermentation resulted in 29.4 g/L succinic acid concentration with yield of 0.56 g/g and productivity of 0.89 g/L/h (Table 3). In continuous cultures, the succinic acid production efficiency (21.2 g/L, 0.47 g/g yield and 1.27 g/L/h productivity) achieved at dilution rate 0.06 h^−1^ was used in techno-economic evaluation. Fed-batch fermentations led to 34.3 g/L succinic acid concentration with a yield of 0.5 g/g and a productivity of 0.75 g/L/h. Fed-batch fermentations were not used in the technoeconomic evaluation due to the low productivity achieved.

**Fig. 7:**
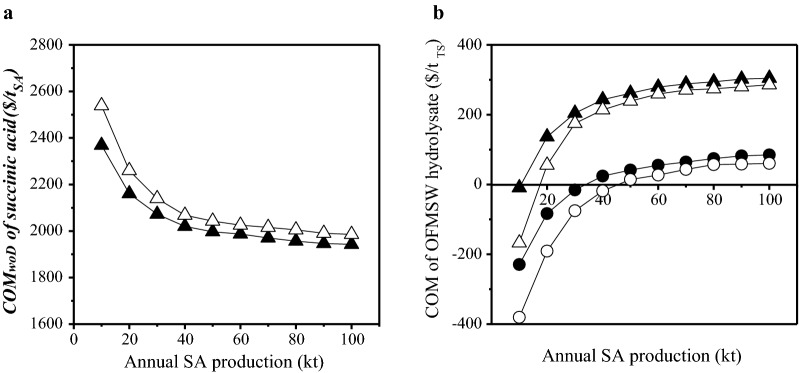
Cost of manufacture of succinic acid ($/t_SA_) at different annual production capacities (**a**) and cost of manufacture of OFMSW hydrolysate per t total sugars ($/t_TS_) at different annual production capacities of succinic acid (**b**) for continuous (filled triangle, filled circle) and batch (open triangle, open circle) cultures. The OFMSW hydrolysate cost has been estimated considering two different MSP for succinic acid, namely 2.94 $/kg_SA_ (triangles) and 2.5 $/kg_SA_ (circles)

The design and costing methodology were applied on the fermentation stage including media sterilization and the downstream separation and purification (DSP) stage that included centrifugation, activated carbon treatment, cation exchange resin treatment, evaporation, crystallization and drying unit operations. The estimation of the optimal design of the fermentation stage, the optimal scheduling of unit operations and the cost estimation of unit operations is based on the work presented by Dheskali et al. [41]. The cost of manufacture for succinic acid did not consider the upstream stage of OFMSW pretreatment and hydrolysis. The production cost of OFMSW hydrolysate was subsequently estimated in order to achieve a minimum selling price (MSP) of bio-based succinic acid equal to its market price (2.94 $/kg according to [12]) or a potential market price of 2.5 $/kg at varying annual succinic acid production capacities (Fig. 7). In this way, the profitability potential of succinic acid production could be assessed considering variable production cost for OFMSW-derived carbon sources.

The cost of manufacture for bio-based succinic acid, considering only fermentation and DSP stages, is reduced at a lower rate for both continuous and batch cultures at annual capacity higher than 50,000 t (Fig. 7a). This occurs because economies of scale have been reached. The implementation of continuous cultures leads to slightly lower cost of manufacture than batch cultures. Figure 7b shows the production cost of OFMSW-derived sugars that should be achieved at varying succinic acid production capacities in order to satisfy minimum selling prices of 2.94 $/kg and 2.5 $/kg. This means that if a lower OFMSW-derived sugar production cost than the one presented in Fig. 7b at a specific plant capacity is achieved, then lower MSP than the targeted one could be reached. For instance, if an OFMSW hydrolysate production cost of 230 $/t total sugars is assumed, which is close to the market price of glucose syrup derived through enzymatic hydrolysis of corn starch produced by the wet milling process, then a MSP equal to the current market price of bio-based succinic acid (2.94 $/kg) could be achieved at annual succinic acid production capacity of 50,000 t if batch cultures are used and 40,000 t if continuous cultures are used. A MSP equal to 2.5 $/kg could be achieved at OFMSW hydrolysate production cost of 25 $/t total sugars, when the industrial plant produces annually 40,000 t succinic acid via continuous cultures or 60,000 t succinic acid via batch cultures.

## Conclusion

The OFMSW can be considered as a promising feedstock for the production of bio-based succinic acid. Despite the geographical and seasonal variability in OFMSW composition, the utilization of tailor-made enzymatic cocktails can lead sufficient hydrolysis of OFMSW carbohydrates. Efficient succinic acid production could be achieved in both batch and continuous cultures using OFMSW hydrolysates. Techno-economic evaluation showed that the utilization of OFMSW hydrolysates could lead to significantly reduced succinic acid production cost. Further cost reduction is needed though to improve further process profitability.

## Methods

### Bacterial strains and inoculum preparation

The bacterial strains employed for succinic acid production were *Basfia succiniciproducens* JF 4016 (DSM-22022) and *Actinobacillus succinogenes* 130Z (DSM—22257), which were purchased from the Leibniz Institute DSMZ—German Collection of Microorganisms and Cell Cultures. All microorganisms were preserved in cryopreservation vials at − 80 °C in a medium containing liquid culture and 50% (v/v) pure glycerol. Inoculum preparation was carried out in Erlenmeyer flasks in tryptic soya broth (TSB) containing pancreatic digest of casein (17 g/L), NaCl (5 g/L), papaic digest of soy bean (3 g/L), K_2_HPO_4_ (2.5 g/L) and glucose (2.5 g/L). Inoculum preparation was carried out in an orbital shaker for 16–18 h at 37 °C and 180 rpm agitation.

### OFMSW origin and hydrolysate preparation

**Fig. 8:**
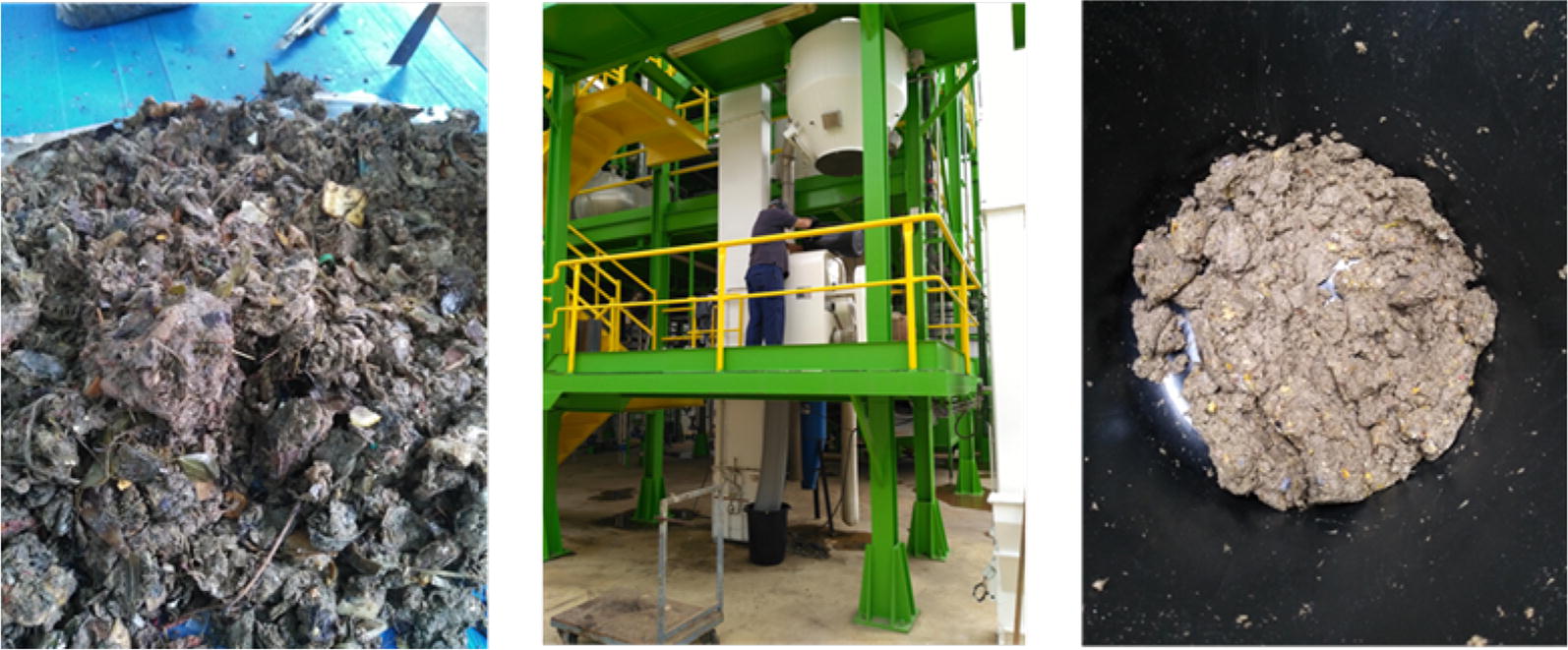
OFMSW sample from MSW treatment plant after inert materials removal (left), milling facilities in IMECAL’s pilot plant (middle), homogenized OFMSW sample after milling (right) ready for characterization and hydrolysis

The following two representative OFMSW streams were collected from an industrial MSW treatment plant in Valencia Metropolitan Area (Spain): (1) “Sorted biowaste” coming from a separate collection in origin from hotels, restaurants, markets and schools (HORECA stream), and (2) “unsorted biowaste” coming from mixed MSW from households bins, after passing different mechanical sorting pre-treatments to recover recyclables and before entering in the biological treatment stage of the plant (composting). Municipal biowaste samples ranging from 50 to 100 kg were collected from the industrial plant in different periods of the year. Once collected from the plant, the remaining inert materials (glass, plastics, stones, textiles, etc.) were removed by manual sorting. After this, the OFMSW samples were mechanically pretreated and milled in a pilot plant hammer mill by the IMECAL company in order to be homogenized and then sterilized by autoclaving at 121 °C for 1 h before submitting to chemical characterization and enzymatic hydrolysis (Fig. 8).

The enzymatic hydrolysis was carried out at 20% solids concentration at 50 °C and pH 5 for 72 h at 150 rpm. The used enzyme cocktail, tailor-made for OFMSW hydrolysis, was supplied by Novozymes A/S company. The optimal used cocktail was a mixture of different enzymes able to hydrolyze up to 86% of glucan and xylan content into fermentable glucose and xylose sugars. The OFMSW hydrolysate was centrifuged (9000 rpm, 4 °C, 10 min) to remove the solid fraction.

### Batch, fed-batch and continuous fermentations

The effect of initial sugar concentration (30, 50, 80 g/L) was evaluated in batch fermentations using both OFMSW hydrolysate and synthetic medium containing commercial carbon sources in a similar ratio (sucrose 4.3%, glucose 73.2%, fructose 18.4%, mannose 0.9%, arabinose 1.8%, galactose 0.3% and glycerol 1.2%) as the one contained on average in OFMSW hydrolysate. The latter medium was used as control experiment. The synthetic medium and OFMSW hydrolysate for *A. succinogenes* and *B. succiniciproducens* fermentations were supplemented with 5 g/L yeast extract and 5 g/L MgCO_3_. The synthetic medium was also supplemented with the following mineral medium: 1.16 g/L NaH_2_PO_4_·H_2_O, 0.31 g/L Na_2_HPO_4_, 1 g/L NaCl, 0.2 g/L MgCl_2_·6H_2_O, 0.2 g/L CaCl_2_·2H_2_O.

The effect of yeast extract (5 g/L), corn steep liquor (5 g/L) and different MgCO_3_ concentrations (5, 10 and 20 g/L) on succinic acid production efficiency were evaluated in *A. succinogenes* fed-batch cultures. All fermentations were initiated with ca. 50 g/L initial total sugar concentration. The feeding solution used was a concentrated OFMSW hydrolysate obtained via rotary evaporation with a total sugar concentration of 400 g/L. Feeding was carried out in pulses when the total sugar concentration was reduced to 5–15 g/L.

Continuous *A. succinogenes* culture was initially operated in batch mode for 24 h and then continuous operation was initiated. The medium at the beginning of continuous fermentation contained 30 g/L glucose, 10 g/L yeast extract, 5 g/L MgCO_3_ and minerals (as described in batch cultures). The continuous culture was initially conducted at a dilution rate of 0.02 h^−1^, with a feeding medium that contained 50 g/L glucose, 5 g/L yeast extract and minerals, in order to increase bacterial cell concentration and stabilize bioreactor operation. Three dilution rates (0.02, 0.04, 0.08 h^−1^) were applied using the same glucose-based synthetic medium. The continuous culture was subsequently operated at different dilution rates (0.02, 0.04, 0.05, 0.06, 0.08, 0.1 h^−1^) with OFMSW hydrolysate as feeding medium containing a total sugar concentration of 35–50 g/L. Yeast extract was added (if needed) into the OFMSW hydrolysate used as feeding medium in order to adjust the FAN concentration at around 500 mg/L. The dilution rate is expressed as the ratio of the volumetric flow rate of the feeding medium to the working volume of the bioreactor. Each dilution rate lasted for approximately 7–8 hydraulic retention times (HRT).

Batch, fed-batch and continuous fermentations were carried out in 1 L bench-top bioreactor (Labfors 4, Infors HT) with 0.5 L working volume. Fermentation pH was controlled at 6.7 with 5 M NaOH. Inoculum size was 10% (v/v). The temperature and agitation were maintained at 37 °C and 100 rpm throughout fermentation. Continuous supply of CO_2_ gas at a flow rate of 0.5 vvm was applied. Carbon and nitrogen sources were sterilized separately from the rest of the medium at 121 °C for 20 min prior to fermentation. Fermentation samples were observed under the microscope to ensure that there was no contamination during fermentation.

### Analytical methods

Bacterial mass concentration was determined by measuring optical density at 660 nm using a spectrophotometer (U-2000 Hitachi). IP was determined according to [42] and FAN was determined according to [43].

To eliminate excess of MgCO_3_, 7% HCl (v/v) solution was added into each fermentation sample. Sugars, organic acids and potential fermentation inhibitors (e.g., furfural, 5-hydroxymethylfurfural or 5-HMF) were determined using a Shimadzu HPLC system with a Shimadzu RI detector and a Rezex ROA-Organic acid H^+^ column. The temperature of the column was 65 °C and the mobile phase was a 10 mM H_2_SO_4_ aqueous solution at 0.6 mL/min flow rate. Monosaccharides were also determined with a Shodex SP0810 (8.0 × 300 mm) column using a Shimadzu HPLC system and Shimadzu RI detector. The temperature of the column was 80 °C and the mobile phase was HPLC grade water at flow rate 1.0 mL/min.

### Determination of OFMSW composition

The compositional analysis was performed in duplicate. Humidity was determined by dying at 105 °C until constant weight was obtained and the ash content by kiln calcination at 575 °C. Samples were air-dried at 40 °C to a moisture content of about 10% and then milled using a centrifugal mill to 1 mm particle size. Carbohydrate content (as glucans and xylans) was determined after a two-step acid hydrolysis according to Sluiter et al. [44]. Starch content was measured using the Total Starch Assay Kit (Megazyme, Ireland) based on the use of thermostable α-amylase and amyloglucosidase. The protein content was determined by two methods: a) by specific staining and spectrophotometry against bovine serum albumin (bicinchoninic acid assay) using Pierce BCA Protein Assay Kit of Thermo Fisher Scientific and b) by the Kjeldahl method using a Tecator digestor and Foss Tecator Kjeltec 8200 Auto Distillation Unit and a Nitrogen-Protein Factor (NF) of 6.25.

The analysis of the remaining components in OFMSW samples was performed as gravimetric analysis in the following sequential steps. The first step was Soxhlet extraction of fat by processing 10 g material and 500 ml chloroform for 1 h, followed by overnight drying at 75 °C. The second step was extraction of water solubles using 3.0 g of fat free material extracted with water for 0.5 h at room temperature followed by dry filtration and overnight drying at 75 °C and weighed. The third step focused on quantitation of pectin by extraction with 3% EDTA solution, pH 4.0 for 4 h in an 85 °C water bath, followed by 500 ml hot water material wash at 50 °C and overnight drying at 75 °C and weighed. The fourth step focused on quantification of the lignin content as follows: approximately 2.0 g material was stirred in 300 ml water containing 20 mL of 10% acetic acid and 10 g NaClO_2_, in 75 °C water bath for 1 h. A 10 mL of 10% acetic acid and 5 g NaClO_2_ was added after 1 h and the reaction was stopped by placing the sample in ice water. The sample was filtered through a glass filter and washed three times with 100 ml water at 50 °C, two times with 100 ml ethanol aqueous solution (96%) and one time with 100 ml acetone. The filtered sample was dried at 75 °C overnight and weighed.

### Determination of the composition of liquid and solid fractions in OFMSW hydrolysate

Total solids were determined according to Sluiter et al. [45]. pH was measured with Mettler Toledo pH meter. Ash content was determined by treating samples at 575 °C for 4 h [46]. After separation of the liquid and solid fractions by centrifugation, the following analyses were carried out. The liquid fraction of OFMSW was used for the determination of sugars, organic acids, inhibitors, IP and FAN as reported above.

The solid fraction was freeze dried and was treated with hexane for 4 h in a Soxhlet apparatus to determine the lipid content. The protein content was determined by Kjeldahl method. Starch was determined with the total starch assay kit (Megazyme, Ireland) according to AOAC Official Method 996.11 and pectin content was determined according to [47]. The OFMSW freeze dried solid fraction was treated with ddH_2_O and ethanol to determine the extractives according to the analytical protocol reported by Sluiter et al. [48]. The dried material that resulted from this treatment was used for determination of structural carbohydrates according to the analytical protocol reported by Sluiter et al. [44].

### Preliminary techno-economic evaluation

The bio-based succinic acid production cost has been estimated via preliminary techno-economic evaluation (accuracy up to ± 30%). The fermentation stage including sterilization and the DSP have been considered for the estimation of the production cost. The total number of bioreactors required per batch cycle, the volume of each bioreactor and the minimum bioreactor cost were estimated based on the study of Koutinas et al. [49] and Dheskali et al. [41] using the optimal succinic acid production efficiencies in batch and continuous cultures. The DSP used in this study has been presented by Alexandri et al. [50]. The experimental succinic acid crystal purity achieved in this study using the fermentation broth derived from OFMSW-based cultures was more than 99%. Individual DSP stages resulted in similar mass balances to the ones reported by Alexandri et al. [50]. The UniSim software was used for the estimation of mass and energy balances to design and simulate the whole bioprocess. In the case of DSP, the removal of biomass via centrifugation was carried out assuming 100% removal of the microbial mass with 50% moisture content. The biomass-free liquid stream is then treated with activated carbon for decolorization. The mass removed in this step is insignificant, thus only energy requirements were taken into consideration. Treatment with cation exchange resins was subsequently carried out for the removal of Na^+^ and Mg^2+^ and the acidification of organic acid salts into their acid form. Evaporation of the acidified liquid stream is carried out using a mechanical vapor recompression forced circulation evaporator system to produce a concentrated solution of succinic acid (216 g/L). The simulation of the DSP process indicates that 76% of the acetic acid and 89% of the formic acid were removed in the evaporation step. The concentrated succinic acid-rich liquid stream produced via evaporation is processed via crystallization at 4 °C resulting in 84% recovery of succinic acid crystals. In order to increase the succinic acid purity (95%) achieved in the first crystallization step, recrystallization was applied by adding water with the final succinic acid purity exceeding 99%. The liquid stream remaining after crystallization containing the non-precipitated succinic acid was recirculated in the evaporation step in order to reduce succinic acid losses and achieve recovery yields of around 95%. The succinic acid crystals were dried at 70 °C using a spray dryer.

Preliminary techno-economic evaluation has been carried out considering annual plant operation of 8300 h at varying plant capacities. The cost of manufacture for succinic acid production excluding depreciation (COM_woD_) and the discounted cash flow analysis have been estimated using the methodology presented by Koutinas et al. [49]. The following equation has been used for the estimation of COM_woD_ as proposed by Turton et al. [51]:

$${\text{COM}}_{\text{woD}} = 0.18 \cdot {\text{FCI}} + 2.73 \cdot C_{\text{OL}} + 1.23 \cdot \left( {C_{\text{RM}} + C_{\text{UT}} } \right),$$

where FCI is the fixed capital investment, *C*_OL_ is the cost of operating labor, *C*_RM_ is the cost of raw materials and *C*_UT_ is the cost of utilities. The estimation of *C*_OL_, *C*_RM_ and *C*_UT_ has been described by Koutinas et al. [49]. The estimation of FCI is based on the estimation of the equipment purchase cost multiplied with a Lang factor of 5. The equipment purchase cost is estimated by sizing all unit operation using the methodology described by Koutinas et al. [49].

**Fig. 9:**
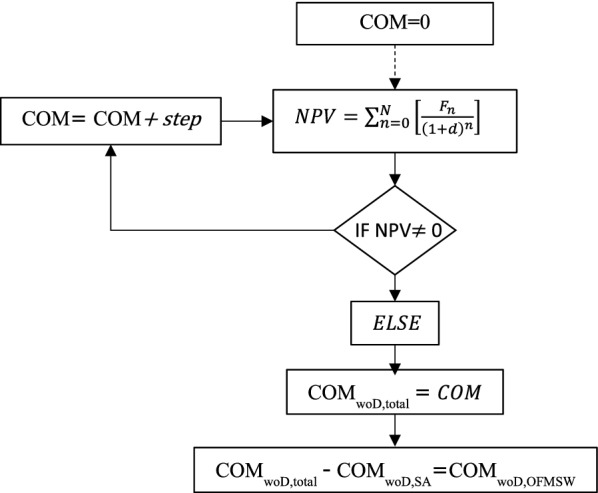
Schematic diagram for the estimation of the OFMSW hydrolysate production cost by iterating the COM_woD,Total_ and calculating the NPV of the process using two different MSP (2.94 $/kg and 2.5 $/kg)

After the estimation of COM_woD_, the discounted cash flow analysis was carried out in order to estimate the production cost of OFMSW hydrolysate per t of sugars considering two MSP for bio-based succinic acid (2.94 $/kg_SA_ and 2.5 $/kg_SA_). The calculations were carried out by iterating the COM_woD,total_ of the whole process (including COM_woD,SA_ for succinic acid production and COM_woD,OFMSW_ for OFMSW hydrolysate production) until the net present value becomes zero at the end of the project life time. The iterating process is illustrated in Fig. 9.

## Data Availability

Not applicable.

## Abbreviations

OFMSW: Organic fraction of municipal solid waste
MSW: Municipal solid waste
YE: Yeast extract
CSL: Corn steep liquor
5-HMF: 5-Hydroxymethylfurfural
HRT: Hydraulic retention time
PEP: Phosphoenolpyruvate carboxykinase
FCI: Fixed capital investment
COM: Cost of manufacture
DSP: Downstream separation and purification
MSP: Minimum selling price
NPV: Net present value
Glu: Glucose
Suc: Sucrose
Fru: Fructose
Arab: Arabinose
Glyc: Glycerol
SA: Succinic acid
LA: Lactic acid
FA: Formic acid
AA: Acetic acid
OD: Optical density
FAN: Free amino nitrogen
IP: Inorganic phosphorous
